# Rethinking latent TB? Think again

**DOI:** 10.5588/ijtldopen.24.0336

**Published:** 2024-08-01

**Authors:** R.E. Chaisson, P.C. Hopewell

**Affiliations:** ^1^ Johns Hopkins University Center for Tuberculosis Research, Baltimore, MD, USA;; ^2^ University of California, San Francisco, CA, USA

**Keywords:** TB infection, TBI, tuberculin skin testing, interferon-gamma release assay, HIV

Over the past several years, Marcel Behr and colleagues have published a series of commentaries attempting to redefine latent TB. They have done so by refuting the assertions that immunoreactivity to *Mycobacterium tuberculosis* antigens by the tuberculin skin test (TST) or interferon-gamma release assay (IGRA) necessarily represent a viable infection and that infection with tubercle bacilli is lifelong if not treated with antibiotics.^1–3^ Both of these ‘straw man’ arguments have long been known not to be true, and refuting them again does not change our understanding of latent TB. Their most recent essay states that ‘understanding TB epidemiology is the cornerstone of understanding the biology of this infectious killer,’ but their attempt to redefine the natural history of *Mycobacterium tuberculosis* infection is based on a fundamental misunderstanding of epidemiology.^1^ The thrust of their argument is that progression from TB infection (TBI) to disease largely occurs in the 2 years following infection and that subsequently focusing attention and resources on people with positive TSTs or IGRAs is unnecessary. We fundamentally disagree: our arguments against this are as follows.

Most people who are infected with *M. tuberculosis* will never develop disease, even in the absence of preventive treatment.^4^ It has long been known that the greatest incidence of progression from TBI to disease is within the first 2 years following infection, and thereafter rates decline substantially. In the absence of a gold standard test for TBI we are forced to rely on immunologic tests that measure T-cell mediated responses to TB antigens. Although some people may completely clear their infections, many do not. Long-term follow-up of children and adolescents with a positive TST confirm higher rates of disease within 2 years after infection, but cases continue to arise over 20 years, albeit at a much lower rate.^5^ Studies of people of all ages with a positive TST or IGRA consistently show a substantially increased risk of TB, demonstrating that tubercle bacilli remain viable for many years.^6^

Behr and colleagues mistakenly hypothesize that if infection were truly lifelong there would be a resurgence of disease with age-related loss of immune control. In fact, there is an apparent increased rate of TB in older age groups, but the increase does not relate to loss of immune control. This apparent increase in rates among older people was shown definitively a half-century ago by Comstock to be the result of a cohort effect, not a resurgence of infections late in life.^7^ Rates of TB are consistently higher in older age groups because the prevalence of infection acquired earlier in life is higher: older persons had more opportunity to become infected. Countering this trend, over the years following infection there is a steady decrease in the risk of TB.^8^ The result of the cohort effect is shown in Figure 1. In 1972, older age groups had the highest rates of TB in the United States, but one to two decades earlier the same cohorts had substantially higher incidence rates.^7^ It is therefore clear that disease can occur even decades after becoming infected, though most people will never develop active TB. For this to occur, tubercle bacilli must remain viable and replication competent for years following infection.

**Figure 1.**
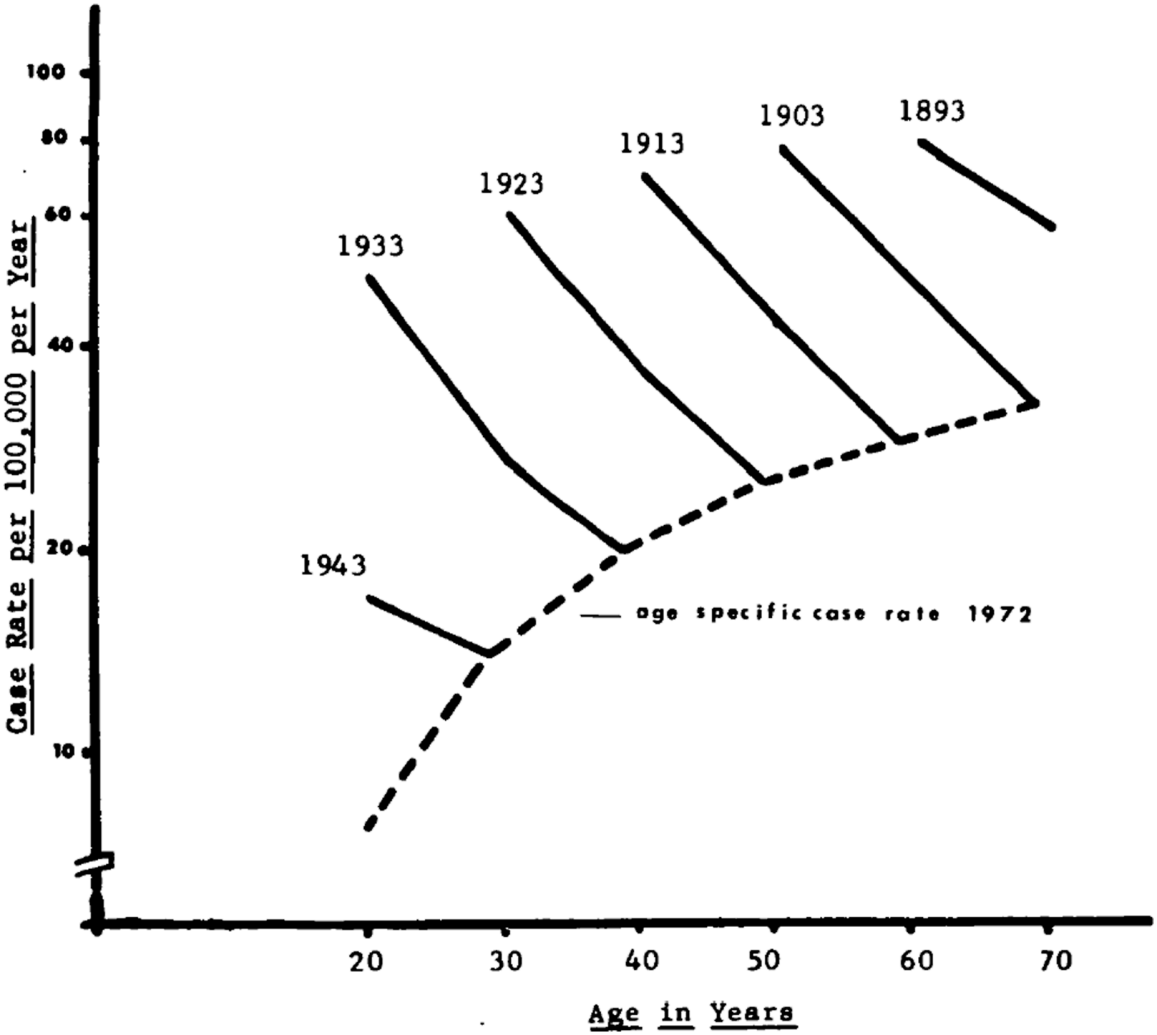
Age-specific incidence rates of TB in the United States in 1972. The dotted line shows rates per 100,000, with increasing incidence in older individuals. The solid lines show the incidence rates in each age cohort over the previous 20 years, demonstrating sharp declines in incidence as the cohort ages. The data support the hypothesis that, while incidence declines with age, the higher prevalence of latent infection in older cohorts results in a higher age-specific incidence (reprinted from Ref. #7 with permission from Oxford University Press).

The important epidemiologic principle that is misunderstood by Behr and colleagues is that large numbers of episodes of TB can occur in people with remote infections^*^ because there are so many of them with TBI. Rather than a resurgence of TB in older individuals, what we see is seepage: a low rate of disease in a very large population. As Comstock wrote, ‘Even those who pass through the highest period of risk…still have a lifetime risk which may actually exceed the initial risk because of the cumulative effect of a low risk operating over many years.’^7^ He subsequently noted, ‘Although we cannot identify these unfortunate persons at the present time, we do have clues that will allow us to concentrate our efforts where the benefit-risk ratios are the greatest.’^9^ In the US and globally, older populations continue to experience the highest TB incidence rates; in the US, the majority of foreign-born individuals with TB emigrated more than five years previously, and 36% have been in the US >20 years.^10^ Half of the risk of TB occurs more than 2 years following infection in these individuals from high-burden settings. As shown in Figure 2, global incidence rates of TB peak at ages 55–64 years and remain high thereafter.^11^

**Figure 2.**
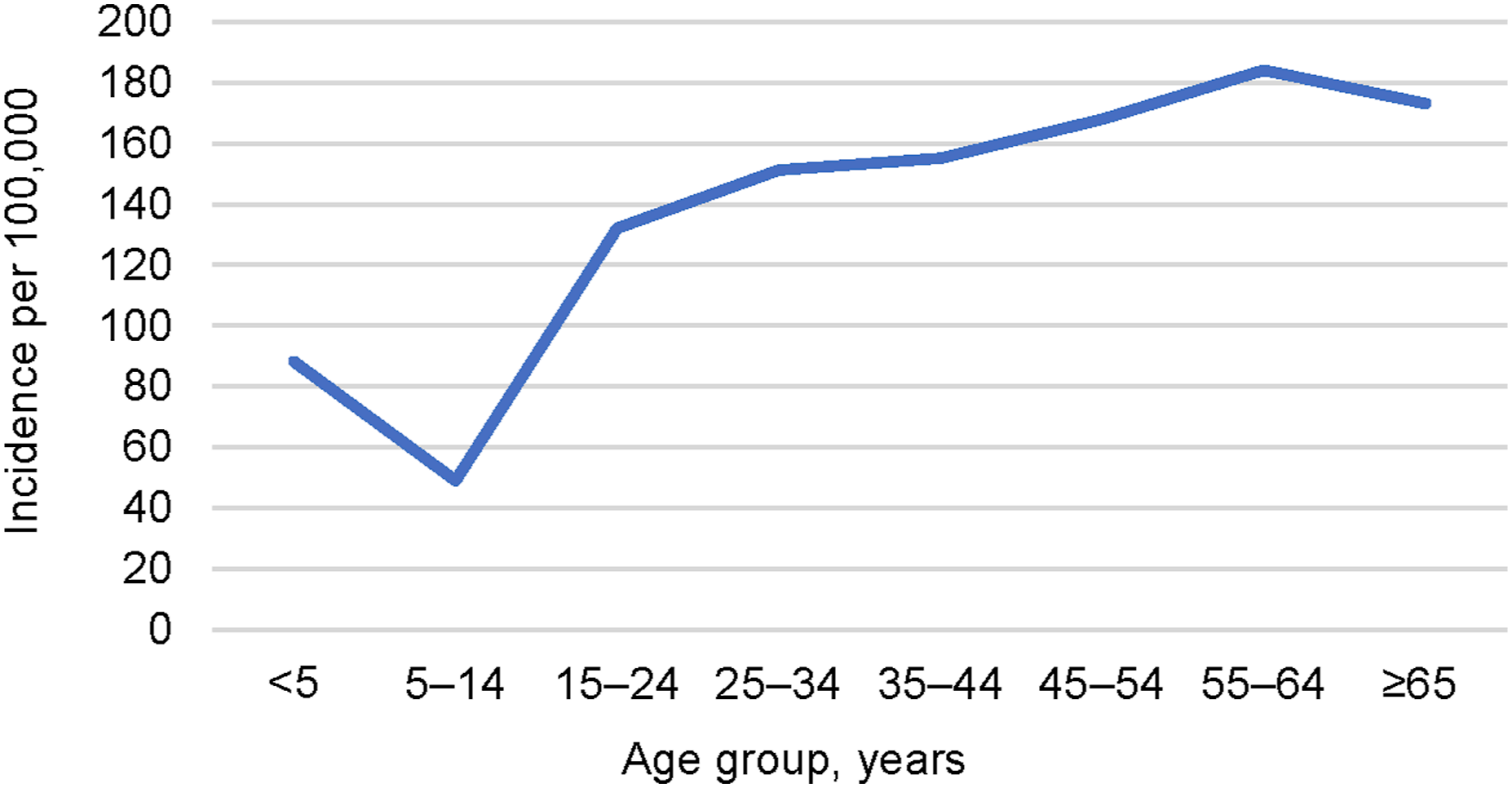
Global estimated age-specific incidence rates of TB per 100,000 people in 2022, demonstrating rising rates of disease associated with aging (data from^11^).

Public health measures to control TB are focused on reducing the risk of disease in those population groups at highest risk. No one has ever suggested that we should try to treat everyone who has a reactive TB test with antibiotics, as Behr and colleagues imply. They attempt to support their assertion that most people self-cure their infection by citing rates of TB in people with HIV, recipients of TNF-alpha inhibitors and transplant recipients, all of whom have substantially increased rates of disease, even in cohorts where the prevalence of positive TB tests is likely low. They contend that because >90% of these populations do not develop TB, focusing prevention efforts on them is not necessary. However, it is important to note that even a 1% incidence of TB correlates to 1,000/100,000, a population risk that is considered extremely high. The CDC, WHO and the Union have consistently recommended targeted testing and treatment for those individuals at the highest risk of developing active TB.^12–14^ As Behr et al. state, recently infected people (such as household contacts of TB patients), are one such important group, but people with HIV infection, those undergoing immunosuppressive therapy, patients with radiographic abnormalities suggesting quiescent disease, and young children with positive TB tests should also be treated. The relative risk of TB in someone with HIV infection and a positive IGRA is 11-times higher than in those without a positive test, and for people receiving immunosuppressive therapy the relative incidence is 48-times higher.^6^ In populations with remote infections, isoniazid and other preventive therapies, which require metabolically active bacilli to work, are effective.^15^ In addition to the public health benefits of preventing TB in these groups, there is tremendous personal benefit. Similarly, vaccine trials for previously infected people are targeting adolescents and young adults with relatively high rates of progression, and not the entire global population of people with positive tests.^16^

Given the poor predictive value of immunologic tests for progression to active TB disease, research on biomarkers to identify those at greatest risk and who would benefit from preventive treatment has been a priority for many years.^17^ Although targeting preventive interventions to those epidemiologic groups at highest risk for progression to disease is currently our best strategy, it would be preferable to have biomarkers that better predict risk. Research to identify such assays should be, and are, a high priority. Understanding the epidemiology of TB is essential, but ignoring the reality that many infections are long-lived is not an evidence-based approach.

## Footnotes

* A remote infection refers to a previous TB infection that has been contained by the person's immune system
